# Isopentyl-Sulfide-Impregnated Nano-MnO_2_ for the Selective Sorption of Pd(II) from the Leaching Liquor of Ores

**DOI:** 10.3390/molecules22071117

**Published:** 2017-07-06

**Authors:** Shengjie Wu, Mingjin Xie, Qin Zhang, Lijiang Zhong, Muhan Chen, Zhangjie Huang

**Affiliations:** School of Chemistry Science and Engineering, Yunnan University, Cuihu North Road No. 2, Kunming 650091, China; 12015001003@ynu.edu.cn (S.W.); mjxie@ynu.edu.cn (M.X.); 12016000408@ynu.edu.cn (Q.Z.); 12016000390@ynu.edu.cn (L.Z.); 12015001006@ynu.edu.cn (M.C.)

**Keywords:** manganese dioxide, isopentyl sulfide, palladium, adsorption, separation

## Abstract

Conventional separation methods are not suitable for recovering palladium present in low concentrations in ore leaching solutions. In this study, a novel isopentyl sulfide (S_201_)-impregnated α-MnO_2_ nanorod adsorbent (BISIN) was prepared, characterized, and applied for the selective adsorption and separation of palladium from the leaching liquor of ores. Batch studies were carried out, and the main adsorption parameters were systematically investigated, in addition to the relevant thermodynamic parameters, isotherms, and kinetic models. The thermodynamic parameters reflected the endothermic and spontaneous nature of the adsorption. Moreover, the experimental results indicated that the Langmuir isotherm model fits the palladium adsorption data well and the adsorption was well described by the pseudo-second-order kinetic model. The main adsorption mechanisms of palladium were elucidated at the molecular level by X-ray crystal structure analysis. Thiourea was found to be an excellent desorption agent, and the palladium-thiourea complex was also confirmed by X-ray crystal structure analysis. The results indicated that almost all of the Pd(II) (>99.0%) is adsorbed on BISIN, whereas less than 2% of the adsorbed Pt(IV), Fe^3+^, Cu^2+^, Ni^2+^, and Co^2+^ is observed under the optimum conditions. The proposed method can be used for the efficient adsorption and separation of palladium from the leaching liquor of ores.

## 1. Introduction

Conventionally, chemical precipitation and solution extraction have been employed in industry for the separation and recovery of palladium from the leaching liquors of ore or spent automotive catalysts [1,2,3,4]. Until the mid-1970s, various companies widely employed chemical precipitation for the separation and recovery of platinum-group metals [5]. Chemical precipitation involves a series of precipitation processes, which exhibit slow kinetics, low selectivity, and high chemical consumption. In China, palladium is a scarce resource which is extracted as a by-product from Cu–Ni sulfide ores. The content of palladium in the Cu–Ni sulfide ores is less than 1 g·t^−1^ [6]. Hence, generally, the concentration of Pd(II) in the leaching solutions of ores in the industry is extremely low, only 10 mg·L^−1^ to 45 mg·L^−1^. Owing to these low Pd(II) concentrations, solvent extraction must be performed at a high phase ratio. However, traditional extraction equipment is not suitable for this process. The prerequisite for employing solvent extraction is that the concentration of palladium in the solution must be greater than several hundreds of milligrams per liter [7]. Therefore, palladium in the solution must be preconcentrated to an acceptable level before it can be separated by solvent extraction. The enrichment factor depends on the ore grade and leaching process parameters. All these factors make extraction of palladium from leaching solutions a challenging task because of the low Pd(II) concentrations and complicated matrices. Some additional methods that have been developed to enrich and separate palladium include membrane separation [8], ion exchange [5], and adsorption [9,10,11,12].

Among these methods, adsorption is optimum for removing palladium ions from dilute aqueous solutions. Several sorbents have been used to recover palladium, e.g., activated carbon [13,14], bioadsorbents [15,16,17,18], and functionalized silica [19]. However, most of these adsorbents exhibit poor adsorption selectivity, which limits their practical applications. They are not suitable for the separation of palladium ions from the complex leaching solution of ores.

In recent years, nano-MnO_2_ has attracted attention because of its high specific surface area, unique aberrant octahedron porous structure, and morphology, enabling a high adsorption capacity [20,21]. The sulfur atom is considered a soft Lewis base and exhibits a strong affinity for soft Lewis acids such as Pd(II), Ag(I), and Hg(II). On the other hand, Ni^2+^, Cu^2+^, Fe^3+^, and Co^2+^ are hard Lewis acids, which cannot be adsorbed by soft sulfide bases [22]. Generally, sulfides are used as extractants for the solvent extraction of palladium from complicated solution matrices [23]. Compared to solvent extraction, solid-phase separation is considerably better for removing Pd(II) from dilute aqueous solutions. In this study, isopentyl sulfide (S_201_) was successfully anchored on α-MnO_2_ nanorods, affording a stable bis(isopentyl)-sulfide-impregnated α-MnO_2_ nanorod adsorbent (BISIN). Because of the unique structure of α-MnO_2_-nanorods, the organic S_201_ ligand can enter the interior of α-MnO_2_-nanorods. S_201_ was incorporated into the α-MnO_2_-nanorods by non-specific interactions via hydrogen bonding and van der Waals forces. The high adsorbent stability also led to the subsequent secondary interaction with Pd(II). The S_201_ molecule contains a S donor atom, which can form a strong complex with Pd(II). The triangular interaction between α-MnO_2_ nanorods, S_201_, and Pd(II) is expected to be favorable for enhancing the adsorption of Pd(II) from the solution. 

Thus far, to the best of our knowledge, no study has reported the adsorption and separation of Pd(II) from complicated ore leaching liquors using sulfide-impregnated nano-MnO_2_ adsorbents. In this study, the selective adsorption and desorption performance of this new adsorbent for Pd(II) was investigated from leaching liquors of the Cu–Ni sulfide ores. Key parameters such as optimum pH, initial Pd(II) concentration, sorbent amount, and contact time were examined. The adsorbent was characterized by Fourier transform infrared (FTIR) spectroscopy, scanning electron microscopy (SEM), X-ray diffraction (XRD), and N_2_ adsorption–desorption experiments. In addition, the thermodynamic parameters, isotherms, and kinetic models were investigated.

## 2. Results and Discussion 

### 2.1. Characterization

#### 2.1.1. FTIR Spectra

**Figure 1:**
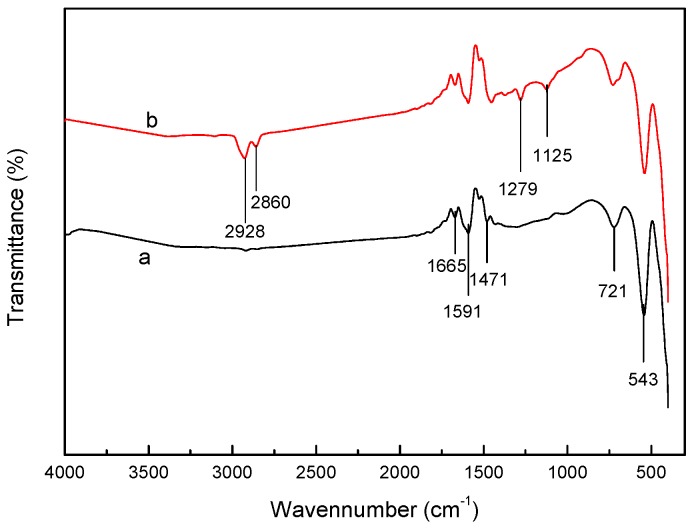
FT-IR spectra of (a) α-MnO_2_ nanorods and (b) BISIN.

Figure 1 shows the FTIR spectra of α-MnO_2_ nanorods and BISIN. Peaks were observed at 721 and 543 cm^−1^ in the spectra of α-MnO_2_ nanorods before impregnation. These peaks are characteristic of Mn–O–Mn vibrations [20]. In addition, peaks were observed at 1665 and 1591 cm^−1^, corresponding to the O–H bending vibration of the –OH groups on the α-MnO_2_ nanorod surface [21]. The strongest absorption peaks of BISIN were observed at 2928 and 2860 cm^−1^, corresponding to the stretching vibrations of the C–H bond in the –CH_3_ and –CH_2_ groups of the alkyl chains, while C–S stretching vibrations were observed at 1279 and 1125 cm^−1^. Thus, the FTIR data in Figure 1 strongly suggested that α-MnO_2_ nanorods are impregnated with S_201_.

#### 2.1.2. XRD Analysis

**Figure 2:**
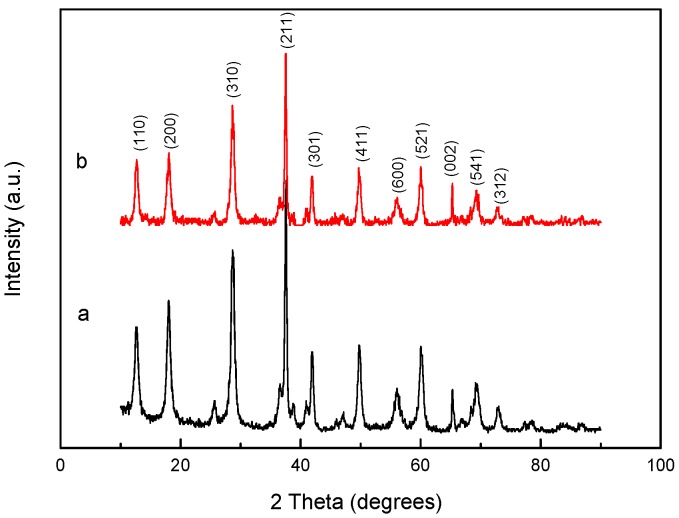
XRD patterns of (a) α-MnO_2_ nanorods and (b) BISIN.

Figure 2 shows the XRD patterns of the synthesized α-MnO_2_ nanorods and BISIN. The peaks at 2θ values in the XRD patterns of the α-MnO_2_ nanorods were consistent with the diffraction data from the standard JCPDS card (no. 44-0141). Moreover, no clear changes were observed in the structure of the α-MnO_2_ nanorods before and after impregnation with S_201_. The results indicated that the functionalization of S_201_ mainly occurs on the α-MnO_2_ nanorod surface [24].

#### 2.1.3. BET Surface Areas and Pore Volumes

**Figure 3:**
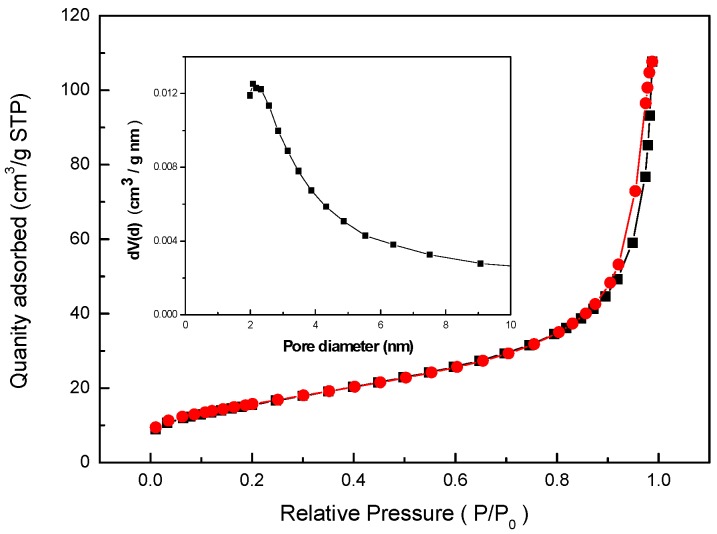
Nitrogen adsorption-desorption isotherm and BJH pore size distribution (inset) of BISIN.

Figure 3 shows the nitrogen adsorption–desorption isotherms of BISIN and the corresponding pore size distributions. A Type IV isotherm was observed, which is characteristic of mesoporous materials. The pore size distributions of the as-prepared BISIN samples were calculated by the Barrett-Joyner-Halenda model. The results indicated that the mean pore diameter of the BISIN sample is 4.9 nm, the total pore volume is 0.12 cm^3^·g^−1^, and the BET surface area of the sample is 56.5 m^2^ g^−1^. The mean pore diameter, total pore volume, and BET surface area of α-MnO_2_ nanorods were 9.8 nm, 0.43 cm^3^·g^−1^, and 101.8 m^2^·g^−1^, respectively. The pore size values of BISIN were clearly less than those of α-MnO_2_ nanorods, possibly because the S_201_ groups inside the tunnels in the crystal lattices of the α-MnO_2_ nanorods block the entrance to some structural channels, thereby decreasing the pore volume [24,25].

#### 2.1.4. SEM Analysis

**Figure 4:**
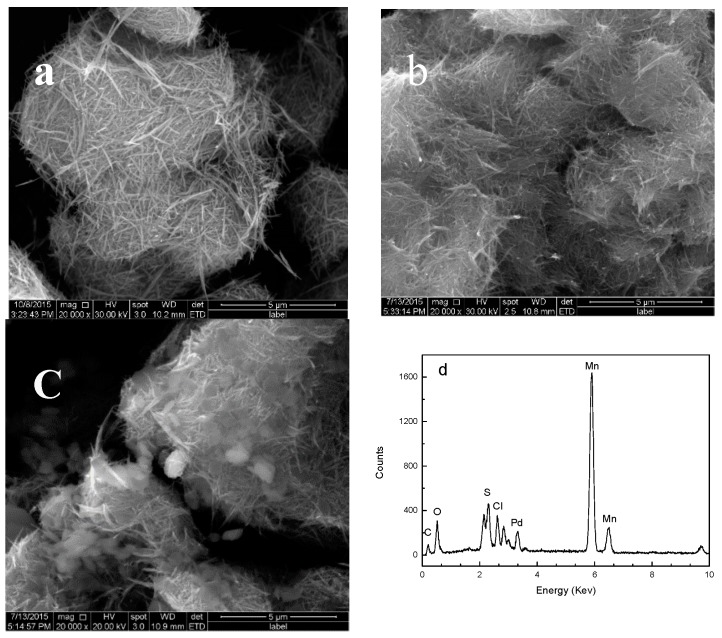
SEM images of (**a**) α-MnO_2_; (**b**) BISIN; (**c**) Pd(II)-BISIN; and EDS spectrum of Pd(II)-BISIN (**d**).

Figure 4 shows the SEM images of the α-MnO_2_ nanorods, BISIN, and Pd(II)-BISIN, as well as the EDS spectrum of Pd(II)-BISIN. Cylindrical α-MnO_2_ nanorods with lengths ranging from 300 to 800 nm and diameters ranging from 25 to 87 nm were observed (Figure 4a). Straight, smooth nanorods exhibited homogenous, flat surfaces. After surface modification with S_201_, the resulting BISIN retained their cylindrical shape. No obvious change was observed in the surface morphology after modification with S_201_, while the structure of BISIN was more compact than that of α-MnO_2_ nanorods (Figure 4b). From the EDS spectrum of Pd(II)-BISIN, the BISIN surface mainly consisted of Mn, O, Pd, S, C, and Cl (Figure 4c,d), indicating that Pd(II) is adsorbed on the BISIN surface. These results confirmed that S_201_ is successfully anchored on the α-MnO_2_ nanorods and that Pd(II) is effectively adsorbed on BISIN.

### 2.2. Effect of pH

**Figure 5:**
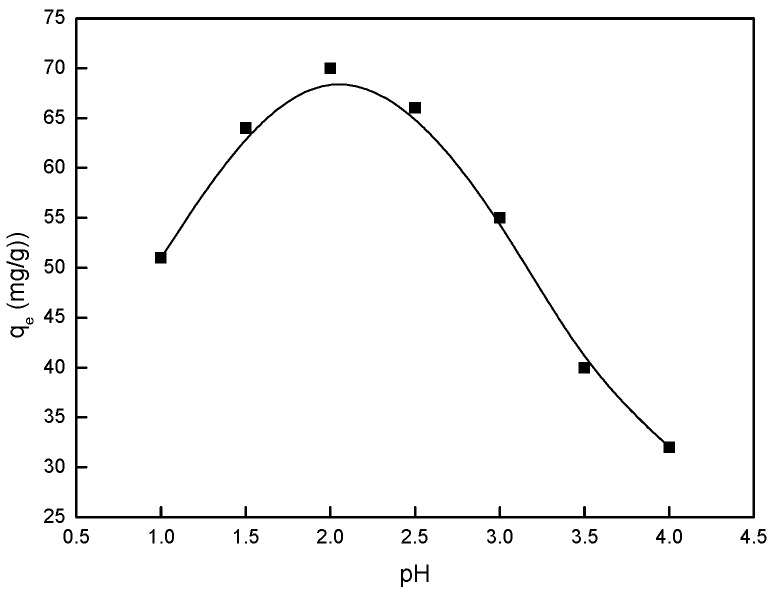
The influence of pH on the adsorption of Pd(II).

The pH of the Pd(II) solution is crucial to adsorption as it can alter the surface charge of BISIN and determine the nature of the Pd(II) species in solution. The optimum pH was determined using solutions of different pH, ranging from 1.0 to 4.0. The initial concentrations of Pd(II) were 50.0 mg·L^−1^, while the BISIN amount was 35 mg, and the solution volume was 50.0 mL at 295 K. Figure 5 shows the effect of pH on the adsorption of Pd(II). With the initial decrease in the pH from 4.0 to 2.0, the equilibrium adsorption capacity (q_e_) increased. However, at a pH of greater than 4.0, the formation of the hydroxyl complexes started, with the primary Pd(II) species being Pd(OH)^+^, Pd(OH)_2_, or Pd(OH)_4_^2−^. Hence, further experiments at a higher pH (>4) are not conducted because of the formation of the palladium precipitates in the solution. At a pH <2.0, the adsorption efficiencies of Pd(II) decreased. At pH = 2.0, the maximum removal efficiency for Pd(II) was achieved. Hence, a pH of 2.0 is selected for all subsequent experiments.

### 2.3. Effect of the Initial Metal Concentration

**Figure 6:**
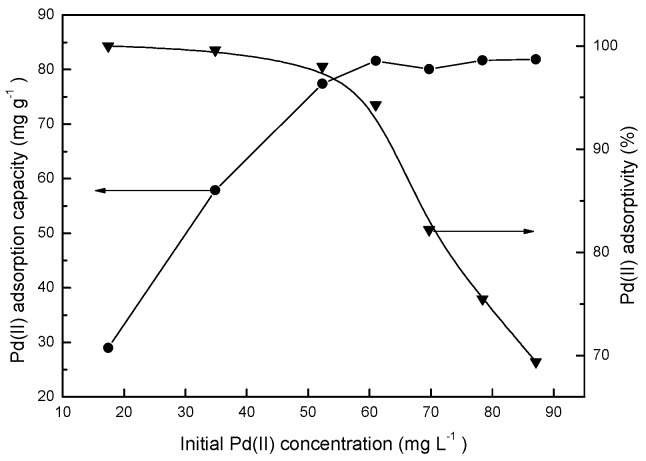
The influence of initial Pd(II) concentration on the adsorption.

Figure 6 shows the effect of the variation in the initial Pd(II) concentration in the aqueous phase on adsorption. Tests were carried out at an initial pH of 2.0 in the aqueous solution, 35 mg of BISIN, 50.0 mL of solution, temperature of 295 ± 1 K, and an adsorption time of 180 min. The higher the initial metal concentration, the lower the percentage of Pd(II) adsorbed (Figure 6). With the increase in the initial Pd(II) concentration from 17.4 to 52.0 mg·L^−1^, the percentage of palladium adsorbed decreased slightly (from 99.9% to 99.2%). With the further increase in the initial Pd(II) concentration from 52.0 to 80.0 mg·L^−1^, the percentage of palladium adsorbed decreased rapidly (from 99.2% to 71.5%). In contrast, the equilibrium adsorption capacity of Pd(II) significantly increased with the increase in the initial Pd(II) concentration. The maximum adsorption capacity was achieved at an initial Pd(II) concentration greater than 80.0 mg·L^−1^. Hence, the adsorbent exhibits high adsorption efficiencies for low Pd(II) concentrations. Pd(II) was almost completely (>99.0%) adsorbed at an initial Pd(II) concentration of less than 52.0 mg·L^−1^. The concentration of Pd(II) in leaching solutions of the Cu–Ni sulfide ores is typically less than 50.0 mg·L^−1^. Thus, the proposed method can be applied for the adsorption of low concentrations of Pd(II) from the leaching solutions of the Cu–Ni sulfide ores in the industry.

### 2.4. Effect of the Sorbent Amount

The effects of the sorbent amount on the adsorption of Pd(II) were investigated by single-component adsorption experiments. The experimental parameters were maintained constant: initial Pd(II) concentration, 50.0 mg·L^−1^; solution volume, 50.0 mL; initial pH of Pd(II) solution, 2.0; adsorption time, 180 min. The results indicated that the sorbent amount remarkably affects the adsorption of Pd(II). The BISIN amount was varied from 10 to 60 mg. With the increase in the sorbent amount from 10 to 35 mg, the percentage of Pd(II) adsorbed increased from 38.5% to 99.1%. With the further increase in the BISIN amount from 35 mg to 60 mg, the percentage of Pd(II) adsorbed was almost constant. Hence, 35 mg of BISIN is used in the following experiments.

### 2.5. Kinetic Model

**Figure 7:**
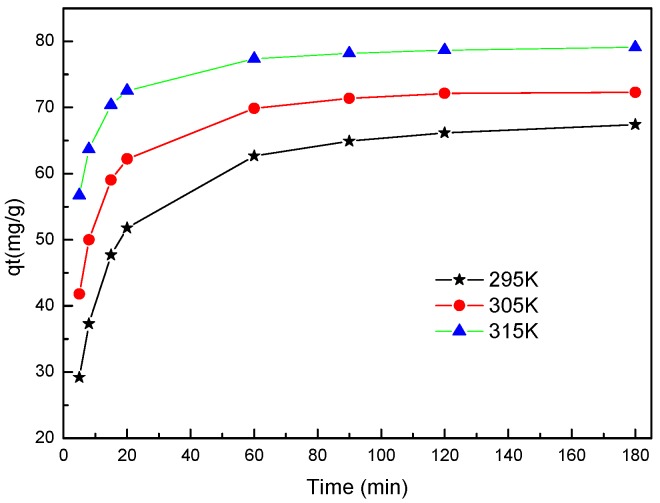
Effect of contact time and temperature on the adsorption rates of Pd(II) onto BISIN.

The kinetics for the adsorption of Pd(II) on the adsorbent were examined by single-component adsorption experiments. Figure 7 shows the adsorption rates of Pd(II) on BISIN at 295 K, 305 K, and 315 K. For all of these experiments, the initial Pd(II) concentration was 50.0 mg·L^−1^, the BISIN amount was 35 mg, and the solution volume was 50 mL. The pH of the solution was adjusted to 2.0 by the addition of a dilute NaOH or HCl solution. From Figure 7, a rapid adsorption rate was observed in the initial 30 min, after which it slowed down and approached equilibrium, possibly because of the initial adsorption reaction occurring on the BISIN surface. Subsequent adsorption likely occurred when palladium entered the interior of tunnels and cavities in manganese dioxide. Hence, with increasing adsorption time, the high activity sites on the BISIN surface are first saturated, followed by the diffusion of the adsorbed amount to the interior of BISIN. In addition, temperature significantly affected the adsorption. With increasing temperature, the adsorption capacity also increased.

In this study, the pseudo-first-order, pseudo-second-order, and intraparticle diffusion kinetic models were employed to examine the experimental data for the adsorption of Pd(II) on BISIN. Table 1 summarizes the experimental results for the kinetic parameters obtained for Pd(II) adsorption.

The pseudo-first-order equation is represented as follows:

(1) $$\mathrm{lg}(q_{e}-q_{t})=\mathrm{lg}q_{e}-\frac{k_{1}t}{2.303}$$

where, *q_e_* (mg·g^−1^) and *q_t_* (mg·g^−1^) represent the amounts of Pd(II) adsorbed at equilibrium and time *t*, respectively, and *k*_1_ is the pseudo-first-order constant (min^−1^). The slopes and intercepts of the plots of lg (*q_e_* − *q_t_*) versus t were used to determine the *k*_1_ and *q_e_* values. The correlation coefficients obtained for the pseudo-first-order model at all of the studied temperatures were not satisfactory. Moreover, the *q_e_* values computed from the pseudo-first-order model were different from the experimental *q_e_* values. Therefore, the pseudo-first-order model does not fit the experimental data for the Pd(II)–BISIN adsorption system.

The pseudo-second-order equation is expressed as follows:

(2) $$\frac{t}{q_{t}}=\frac{1}{k_{2}q_{e}{}^{2}}+\frac{t}{q_{e}}$$

Higher correlation coefficients (≥0.9980) were obtained for the pseudo-second-order model, and the calculated *q_e_* values were also in good agreement with the experimental data. Hence, the adsorption kinetics well fit with the pseudo-second-order kinetic model. Chemical sorption is likely the rate-limiting step of adsorption [26].

The third kinetic model evaluated is the intraparticle diffusion model, which is expressed below as Equation (5):

(3) $$q_{t}=k_{p}t^{1/2}+C$$

where *q_t_* (mg·g^−1^) is the amount of Pd(II) adsorbed at time *t*, *kp* is the intraparticle diffusion rate constant (mg·g^−1^ min^1/2^), *C* (mg·g^−1^) is the boundary layer thickness, and t is the contact time (min). By plotting a graph of *q_t_* versus *t*^1/2^, the *kp* and *C* values were determined from the slope and intercept values, respectively. From Table 1, poor correlation coefficients (0.4962−0.6621) were obtained for the intraparticle diffusion model at the three temperatures, indicating that the intraparticle diffusion mode is not the dominant mechanism for the adsorption of Pd(II) on BISIN [27].

### 2.6. Isotherm Models

**Figure 8:**
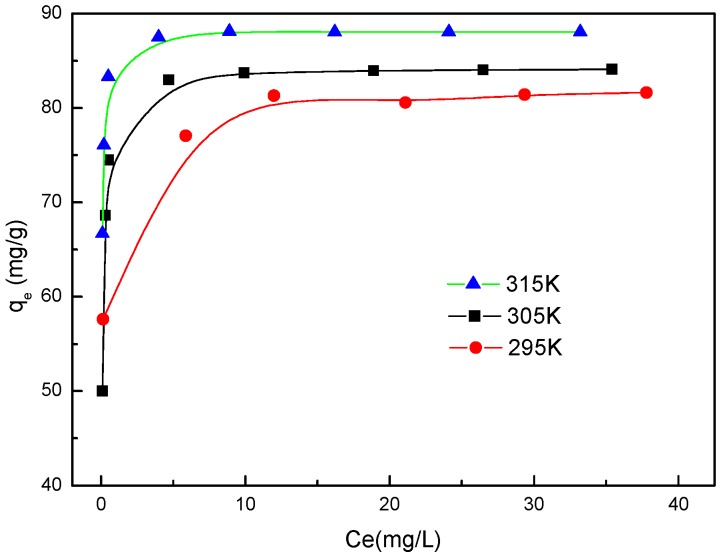
Adsorption isotherms of Pd(II) at different temperatures.

Figure 8 shows the adsorption isotherms of BISIN for Pd(II) at 295 K, 305 K, and 315 K. The initial Pd(II) concentrations were varied between 35.0 and 100.0 mg·L^−1^, while the BISIN amount and solution volume were 35 mg and 50.0 mL, respectively. The solution pH was adjusted to 2.0 by the addition of a dilute NaOH or HCl solution. Batch single-component adsorption experiments were carried out. The *q_e_* of Pd(II) increased with the increase in the equilibrium concentration of Pd(II) in the solution (Figure 8). In this study, the adsorption was described by the Langmuir and Freundlich models.

The Langmuir model is expressed as follows:

(4) $$\frac{C_{e}^{}}{q_{e}}=\frac{1}{q_{m}b}+\frac{C_{e}}{q_{m}}$$

where *q_m_* is the maximum adsorption capacity (mg·g^−1^), *q_e_* is the amount absorbed on BISIN at equilibrium (mg·g^−1^), *Ce* is the equilibrium concentration of metal in solution (mg·L^−1^), and b is the Langmuir adsorption equilibrium constant (L·mg^−1^).

**Figure 9:**
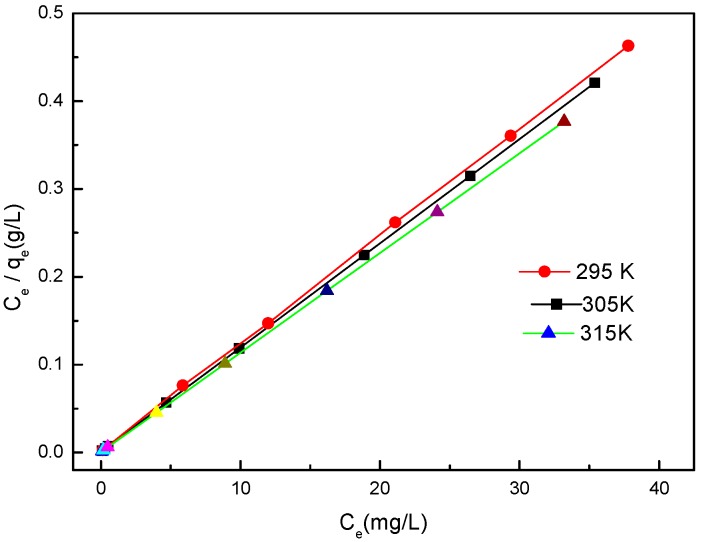
The Langmuir isotherms at different temperatures.

By plotting a graph of *Ce/qe* versus *Ce* (Figure 9), the maximum adsorption capacities of Pd(II) (*q_m_*) can be evaluated from the slope. The *q_m_* values were 81.9, 84.2, and 88.2 mg·g^−1^ at 295, 305, and 315 K, respectively. The experimental results indicated that *q_m_* increases with temperature. This result indicated that the adsorption of Pd(II) on BISIN is an endothermic process.

Table 2 summarizes the experimental data obtained from the Langmuir model for the absorption of Pd(II) on BISIN.

The equilibrium parameter (*R_L_*), also called the dimensionless constant separation factor, helps to predict whether the adsorption is favorable.

*R_L_* can be defined by the following equation:

(5) $$R_{L}=\frac{1}{1+bCo}$$

where *Co* is the initial concentration of Pd(II) in solution (mg·L^−1^), and *b* is the Langmuir adsorption equilibrium constant (L·mg^−1^). The calculated *R_L_* values were found to range from 0 to 1, indicating that the adsorption is favorable [28].

Furthermore, the Freundlich isotherm model was also tested according to Equation (8):

(6) $$\mathrm{lg}q_{e}=\mathrm{lg}K_{F}+\frac{1}{n}\mathrm{lg}C_{e}$$

where *K_F_* is the Freundlich constant (L·g^−1^), and *1/n* is the adsorption intensity.

The linear plots of lg *q_e_* versus lg *Ce* for the adsorption of Pd(II) on BISIN were obtained. Table 2 also summarizes the estimated values for *K_F_, n*, and *R*^2^.

As can be observed from Table 2, the Langmuir equation fitted the experimental data better than the Freundlich equation according to the *R*^2^ values obtained at 295, 305, and 315 K. The high correlation coefficients (*R*^2^ ≥ 0.9991) indicated that the experimental data for adsorption are reasonably well described by the Langmuir isotherm model. In addition, the calculated values of maximum adsorption capacity were similar to the experimental values, further confirming the suitability of the Langmuir isotherm model for fitting the adsorption of Pd(II) on BISIN. The Langmuir equation is based on the assumption that active sites on the absorbent surface are homogenously distributed. In this study, the nano-MnO_2_ surface was covered by the same S_201_ groups, providing the same surface-reactive sites of S atoms coordinating to Pd(II). Therefore, every active site is independent, and a Pd(II) monolayer is adsorbed on BISIN.

Table 3 summarizes the maximal adsorption capacity and selectivity for Pd(II) reported for some adsorbents. The adsorption capacity of BISIN was greater than those of most of the previously reported adsorbents. 

### 2.7. Thermodynamics Studies

To thoroughly investigate the adsorption of Pd(II) on BISIN, the relevant thermodynamic parameters of adsorption were calculated. Specifically, the Gibbs free-energy changes (*∆G*), entropy change (*∆S*), and enthalpy change (*∆H*) were calculated by the following equations: [36]

(7) $$Kc=\frac{qe}{Ce}$$

(8) $$\ln Kc=-\frac{\Delta H}{RT}+\frac{\Delta S}{R}$$

**Figure 10:**
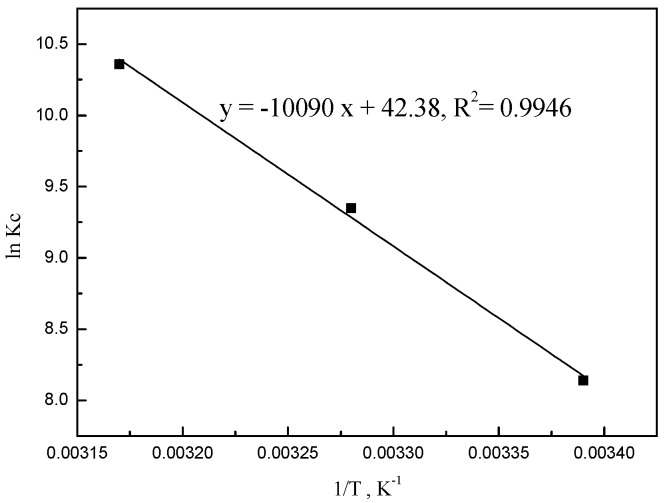
Plot of ln *K_c_* versus *1/T* for adsorption of Pd(II) onto BISIN.

(9) ΔG=ΔH−TΔS

where *Kc* is the thermodynamic equilibrium constant, *q_e_* and *Ce* are the equilibrium adsorption capacity and equilibrium concentration of palladium solution (mol·L^−1^), respectively. By plotting a graph of ln *Kc* versus *T*^−1^, the *∆H* and *∆S* values were calculated from the slope of the straight line and intercept, respectively (Figure 10).

The *∆G* values were −20.1, −23.6, and −27.1 kJ·mol^−1^ at 295, 305, and 315 K, respectively (Table 4), indicating that the reaction is spontaneous. Moreover, the degree of spontaneity for adsorption increased with temperatures. Hence, the increase in temperature is favorable for adsorption. Positive *∆H* (83.9 kJ·mol^−1^) confirmed that the adsorption was endothermic, while positive *∆S* indicated increased randomness at the BISIN/solution interface during the adsorption of palladium on BISIN. At a sufficiently high temperature, *T∆S* would be greater than *∆H* (*∆G* < 0). As shown in Table 4, *∆G* decreased with increasing temperatures, indicating that adsorption is more spontaneous at high temperatures. Shen et al. [37] have suggested that *∆H* for chemisorption is greater than 40 kJ·mol^−1^. Thus, the *∆H* in Table 4 indicated that chemisorption is possibly the rate-controlling step for the adsorption of Pd(II) on BISIN.

### 2.8. Desorption

Batch elution experiments were carried out to evaluate the optimum eluting agent to recover the adsorbed palladium from BISIN. In a typical experiment, 50 mL of a 50 mg·L^−1^ Pd(II) ion solution was adsorbed on 35 mg BISIN. Then, 0.2 M thiourea (5.0 mL) was used as the eluting reagent, and the combined solution was subjected to shaking for 10 min. After elution was completed, the solution was centrifuged for 5 min at 3000 rpm. The percentage of Pd(II) eluted was greater than 98.5% through this process. The Pd(II) ions were released from the BISIN into the thiourea solution under the constant conditions.

### 2.9. Reusability of the Adsorbent

Stability is one of the key factors for evaluating the adsorbent performance. In this study, BISIN was reused up to several times without the significant loss of adsorption. The maximum loss in the adsorption capacity after performing seven adsorption and desorption cycles did not exceed 5%. In the 20th cycle of operation, the adsorption capacity decreased to 62% of the maximum value, corresponding to the loss of S_201_. The reimpregnation of S_201_ into nano-MnO_2_ is a simple process. The experiments indicated that the adsorbent can be regenerated by the reimpregnation of S_201_. The maximum adsorption capacity of the regenerated adsorbent was similar to that of the initial adsorbent.

### 2.10. Experiments with Actual Leaching Liquor from the Cu–Ni Sulfide Ores

The proposed method was applied for the adsorption and separation of palladium from the actual leaching liquor from the Cu–Ni sulfide ores in the Yunnan DaLi region of China. The leaching liquor containing Pd, Pt, Fe, Cu, Ni, and Co was evaporated to near dryness, and then the concentrated liquor was diluted to a known volume using 0.1 mol·L^−1^ HCl. The solution acidity was adjusted to the optimum pH, and Pd(II) was effectively adsorbed on the BISIN adsorbent. Batch multicomponent adsorption and elution experiments were carried out at room temperature (295 ± 1 K). Table 5 summarizes the results obtained.

Based on the experimental data in Table 5, the obtained separation factors (β_Pd/M_) [38,39] ranged from 6.08 × 10^3^ to 2.19 × 10^4^. Greater than 99.0% of Pd(II) was effectively adsorbed on BISIN in the presence of the other metal ions, while less than 2% of Pt(IV), Fe^3+^, Cu^2+^, Ni^2+^, or Co^2+^ was adsorbed on BISIN, indicating palladium is efficiently adsorbed and separated by BISIN from a multicomponent leaching liquor containing Pt(IV), Fe^3+^, Cu^2+^, Ni^2+^, and Co^2+^.

### 2.11. Discussion of Adsorption and Desorption Mechanisms

#### 2.11.1. Adsorption Mechanism

The unique tunnels in the α-MnO_2_ crystal lattices and the high surface area permit the exposure of α-MnO_2_ to a high amount of adsorption sites for S_201_, leading to the high stability of the S_201_ coating. S_201_ penetrated into the tunnels and cavities of α-MnO_2_, as well as anchored on the α-MnO_2_ surface.

The O–H···S hydrogen bonds between the hydroxyL·groups on the nano-MnO_2_ surface and the S atom present in S_201_ are also vital for the stabilization of BISIN [40]. The combination of the unique tunnel structure of α-MnO_2_, intermolecular hydrogen bonding, and van der Waals interactions leads to the high affinity between S_201_ and α-MnO_2._ Hence, S_201_ is impregnated into α-MnO_2_ to form a stable BISIN.

**Figure 11:**
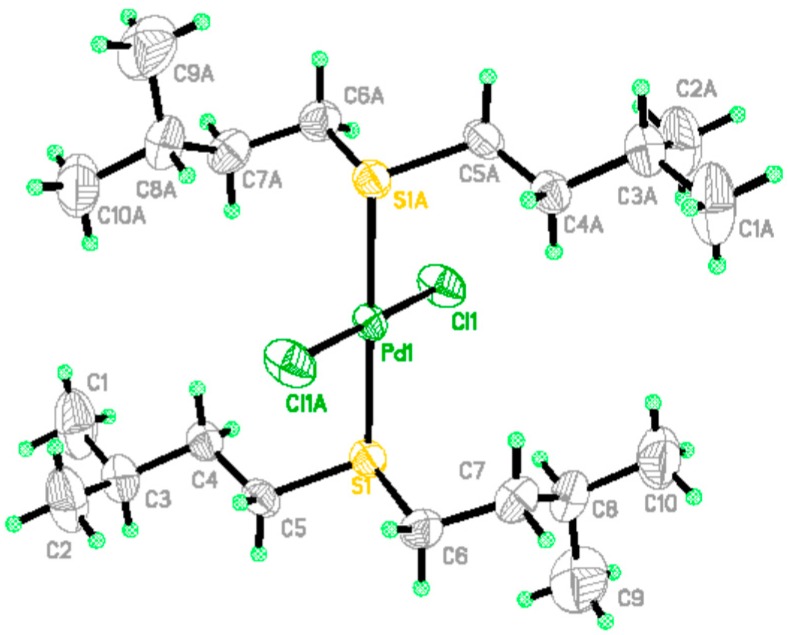
The X-ray crystal structure of PdCl_2_(S_201_)_2_ complex.

The high stability of BISIN led to subsequent secondary interactions with Pd(II). The triangular interaction between nano-MnO_2,_ S_201_, and Pd(II) is expected to be favorable for the enhanced absorption of Pd(II). According to the hard and soft acid and base principle, Pd is a soft acid, which can coordinate to the S atom on BISIN, affording a stable metal chelate. By contrast, Ni^2+^, Cu^2+^, Zn^2+^, and Co^2+^ are hard Lewis acids, which cannot be adsorbed by soft S_201_ bases. Hence, BISIN is used to selectively adsorb palladium ions because of the strong complex formation ability between the S_201_ ligand and Pd(II) ion. Figure 11 shows the X-ray crystallographic structure of the Pd(II)–BISIN adduct.

Table 6 summarizes the crystal data and structural refinements of the complex. The crystallographic data of the complex were deposited at the Cambridge Crystallographic Data Center, CCDC Nos. 1442093, which contains the complete crystallographic data for this paper. The data can be obtained from the Cambridge Crystallographic Data Centre via www.ccdc.cam.ac.uk/. According to the crystallographic data, the S atom exhibits a strong chelating ability with Pd(II). Two S_201_ ligands on the BISIN surface selectively bind to one PdCl_4_^2−^, affording a PdCl_2_(S_201_)_2_ complex (Figure 11). Based on the triangular interaction between nano-MnO_2_, S_201_, and Pd(II), the main mechanisms for the adsorption of palladium on BISIN may be depicted as follows:

(10) $${\mathrm{PdCl}}_{4}{}^{2-}+{\mathrm{MnO}}_{2}\cdot {\left(S_{201}\right)}_{2}\to {\mathrm{PdCl}}_{2}{\left(S_{201}\right)}_{2}\cdot {\mathrm{MnO}}_{2}+2{\mathrm{Cl}}^{-}$$

**Figure 12:**
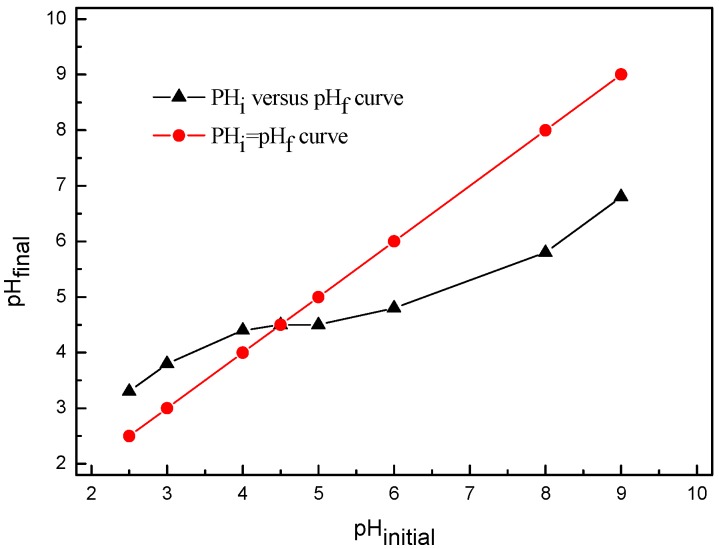
Plot of final pH versus initial pH for determination of pH_pzc._

In addition, the electrostatic interactions play an important role in the sorption of Pd(II) on the adsorbent. To investigate the electrostatic adsorption mechanism, the solution pH was determined, where the charge of the adsorbent surface was zero (pH_pzc_) according to a previously reported method [41]. Figure 12 shows the results. pH_PZC_ is expressed by the point at which the experimental curve (pH_initial_ vs. pH_final_) intersects the straight line given by pH_initial_ = pH_final_. The pH_pzc_ value was 4.5 (Figure 12). At pH < pH_pzc_, the hydroxyL·group and S atom on the BISIN surface were protonated, and the adsorbent surface exhibited positive charges, which would enhance its electrostatic attraction with anion. The Pd(II) solution was predominantly composed of PdCl_4_^2−^ (pH < 4.0), and PdCl_4_^2−^ was adsorbed on the positively charged BISIN surface via electrostatic interactions. Nevertheless, q_e_ decreased at a pH less than 2.0. In this case, because the H^+^ concentration is greater in the solution, the Pd(II) solution composition was changed from PdCl_4_^2−^ to H_2_PdCl_4_. Hence, the electrostatic interactions between the adsorbent and Pd(II) are weakened. Moreover, the protonation of the sulfur atom of the S_201_ group decreased its ability to form chelates with PdCl_4_^2−^ [24]. Thus, the solution pH affects not only the nature of the Pd(II) species but also the distribution of charges on the nano-MnO_2_ surface. A pH of 2.0 was selected for all subsequent adsorption experiments. In addition, a few PtCl_6_^2−^ ions were adsorbed because of the positive charge on the adsorbent surface. On the contrary, metal cations (e.g., Ni^2+^, Cu^2+^, Zn^2+^, and Co^2+^) were repelled by the positively charged adsorbent.

#### 2.11.2. Possible Desorption Mechanism

**Figure 13:**
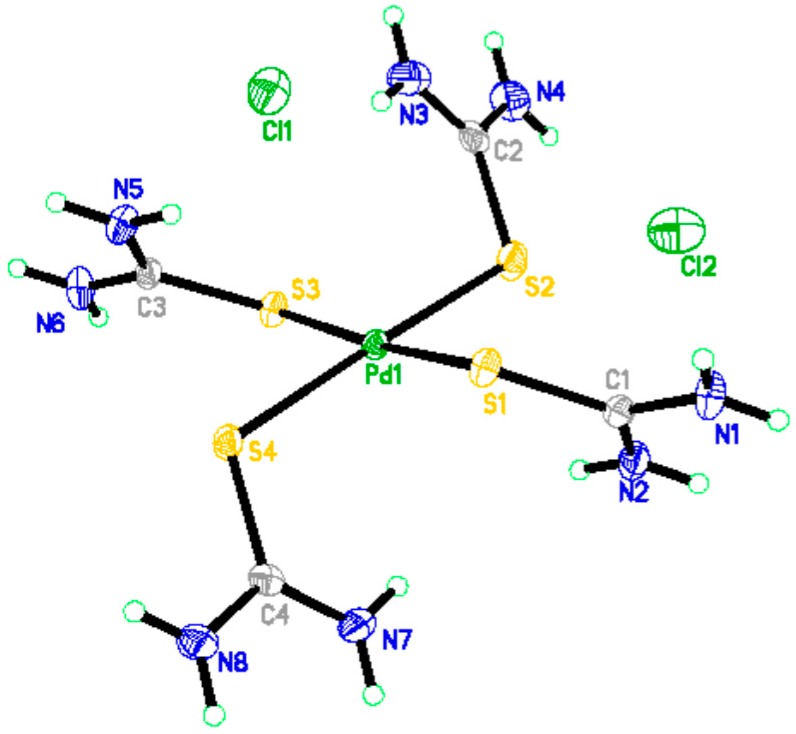
The X-ray crystal structure of thiourea-Pd(II) complex.

The experiments showed that the thiourea solution is a suitable eluent to extract the loaded Pd(II) from the adsorbent. Figure 13 shows the X-ray crystal structure of the thiourea–Pd(II) adduct.

Table 7 summarizes the crystal data and structural refinement of the complex. Crystallographic data of the complex were deposited at the Cambridge Crystallographic Data Center (CCDC Nos. 1417636), contains the complete crystallographic data for this paper. The data are obtained from the Cambridge Crystallographic Data Centre via www.ccdc.cam.ac.uk/. The crystal structure of thiourea–Pd(II) complex indicated that (NH_2_)_2_CS acts as a neutral unidentate ligand coordinating to Pd(II) via the S atom of thiourea (Figure 13). More specifically, Pd(II) coordinated to the four S atoms of four thiourea molecules. According to the crystallographic data, the desorption mechanism of Pd(II) may be depicted as follows:

(11) $${\mathrm{PdCl}}_{2}{\left(S_{201}\right)}_{2}\cdot {\mathrm{MnO}}_{2}+4{{(\mathrm{NH}}_{2})}_{2}\mathrm{CS}\to {\mathrm{Pd}[(\mathrm{NH}}_{2}{)}_{2}{\mathrm{CS}]}_{4}{}^{2+}\cdot 2{\mathrm{Cl}}^{-}+{\mathrm{MnO}}_{2}\cdot {\left(S_{201}\right)}_{2}$$

## 3. Experimental

### 3.1. Reagents and Apparatus

α-MnO_2_ nanorods were prepared by a hydrothermal method. In a typical synthesis, KMnO_4_ (0.35 g), NH_4_H_2_PO_4_ (0.02 g) and NaF (0.15 g)f were added to ultrapure water (50 mL). After continuous sonication for 25 min, the entire mixture was transferred into a 100 mL Teflon-lined autoclave and subjected to hydrothermal treatment at 100 °C for 12 h. The product was collected by centrifugation and washed with ultrapure water and absolute ethanol. S_201_ was provided by Beijing Ruile Kang Separation Technology Corporation Ltd. (Beijing, China). S_201_ was impregnated into the synthesized samples of α-MnO_2_ nanorods via sonication. In a typical adsorbent preparation, nano-MnO_2_ (3.0 g) and S_201_ (30 mL) were added to acetone (50 mL) in an Erlenmeyer flask. After continuous shaking for approximately 15 min using a shaker, the Erlenmeyer flask was transferred to the sonicator, followed by sonication for 30 min. The precipitate was collected by centrifugation, and then the resulting adsorbent sample was washed three times with acetone. Finally, the product was dried in a vacuum oven at 60 °C for 6 h. The leaching liquor of the Cu–Ni sulfide ores and stock solution of Pd(II) (10.00 g L^−1^) were provided by the Yunnan Gold Group Company (Kunming, China). The pH of the leaching liquor was adjusted using a dilute NaOH or HCl solution. All other chemical reagents used herein were of AR grade (Alfa Chemical Industrial Company, Beijing, China). Ultrapure water with a resistivity of 18 MΩ cm was obtained using a UPHW purification device (Ulupure Instrument Corporation, Shanghai, China), which was used to prepare all of the solutions.

An SK5200 sonicator (Kudos Ultrasonic Instrument Company, Shanghai, China) was used to disperse α-MnO_2_ nanorods in solution at an operating power of 500 W. A Z-2000 polarized Zeeman atomic absorption spectrophotometer (Hitachi High-Technologies Corporation, Tokyo, Japan) was utilized to measure the metal ion concentrations. The operating conditions were selected according to the manufacturer recommendations. The pH was measured using a PHS-3C precision pH meter (REX Instrument Factory, Shanghai, China). FTIR spectra were recorded on a Thermo Nicolet 8700 spectrometer (Thermo Fisher Scientific, Waltham, MA, USA) in the wavenumber range of 400–4000 cm^−1^. XRD patterns were recorded to identify the phase structure and composition of the samples over a 2θ range from 10° to 90° using Co Kα radiation. Sample morphologies were observed using an AMRAY 1000B SEM system. Nitrogen adsorption–desorption measurements were carried out at 77 K on a Micromeritics Tristar apparatus (Micromeritics Instrument Corporation, Norcross, GA, USA). Specific surface areas were determined by the Brunauer-Emmet-Teller (BET) analysis. Intensity data for single crystals of the complex were obtained on a SMART 1000 CCD detector (Bruker Corporation, karlsruhe, Germany) with graphite-monochromatized Mo Ka radiation (*λ* = 0.071073 nm).

### 3.2. Batch Adsorption Experiments

Batch adsorption studies were carried out to investigate the adsorption of Pd(II) on BISIN. From batch experiments, the optimum experiment conditions, kinetics, and thermodynamics were investigated.

Typically, a suitable amount of BISIN (35 mg) was added to 250 mL conical flasks containing Pd(II) solutions (50 mL, 50 mg·L^−1^). Subsequently, the sealed conical flasks were placed in a thermostatic shaker for 180 min at 120 rpm and maintained at 295 K. Then, the solutions were centrifuged for 5 min at 3000 rpm. The initial and final concentrations of the metal ions in solution were estimated by atomic absorption spectroscopy. The adsorption efficiency (*E_M_*, %) and equilibrium adsorption capacity (*q_e_*, mg·g^−1^) were calculated by the following equations:

(12) $$E_{M}=\frac{C_{0}-C_{e}}{C_{0}}\times 100\%$$

(13) $$q_{e}=\frac{(C_{0}-C_{e})\times V}{W}$$

where *C*_0_ (mg·L^−1^) is the initial metal ion concentration, *Ce* (mg·L^−1^) is the equilibrium metal ion concentration, *W* is the mass of the added adsorbent (g), and *V* is the volume of the metal ion solution.

## 4. Conclusions

In this study, a new method for the adsorption and separation of palladium using a nano-MnO_2_ adsorbent impregnated with bis(isopentyl) sulfide groups was developed. The optimum pH for the adsorption of Pd(II) by BISIN was determined to be 2.0. The Langmuir isotherm model exhibited higher correlation coefficients of determination compared to the Freundlich isotherm model. The pseudo second-order equation provided the best correlation for the adsorption of Pd(II) on BISIN. More than one mechanism affected the adsorption of Pd(II) on BISIN. Chemisorption was the most important adsorption mechanism as a large number of S atoms on the sorbent exhibited a strong chelating ability with Pd(II). In addition, hydrogen bonding and electrostatic interaction played important roles in adsorption.

The thermodynamic parameters of ∆G < 0 and ∆H > 0 indicated that the adsorption of palladium is a spontaneous and endothermic process. Batch adsorption experiments indicated that BISIN exhibits an excellent adsorption activity, easy regeneration, low cost, high selectivity, and good stability. The results of this study indicated that this new adsorbent can be used for the efficient separation of Pd(II) from complicated leaching solutions of the Cu–Ni sulfide ores.

## Figures and Tables

**Table 1:** Kinetic parameters of Pd(II) adsorption onto BISIN.

Kinetic Parameters	Temperature (*K*)		
	295	305	315
1. Pseudo-first order			
*q_e_*, exp. (mg·g^−1^)	69.2	73.8	79.2
*q_e_*, cal. (mg·g^−1^)	188.4	240.2	169.1
*k*_1_ (min^−1^)	0.016	0.015	0.028
R^2^	0.8921	0.7962	0.9603
2. Pseudo-second order			
*q_e_*, exp. (mg·g^−1^)	69.2	73.8	79.2
*q_e_*, cal. (mg·g^−1^)	70.0	74.5	79.9
*k*_2_ (g mg^−1^·min^−1^)	0.00203	0.00343	0.00609
R^2^	0.9992	0.9991	0.9980
3. Intraparticle diffusion			
*Kp* (mg·g^−1^·min^−0.5^)	2.905	2.213	1.572
*C*	33.37	47.0	61.2
*R*^2^	0.6621	0.5514	0.4962

**Table 2:** Langmuir and Freundlich isotherm parameters of BISIN.

T (K)	*q_m_* ^a^ (mg·g^−1^)	Langmuir Isotherm			Freundlich Isotherm		
		*q_m_* ^b^ (mg·g^−1^)	*b* (L·mg^−1^)	*R*^2^	*K_f_* (L·g^−1^)	*n*	*R*^2^
295	81.4	81.9	5.05	0.9992	66.8	14.5	0.9531
305	83.7	84.2	14.6	0.9991	89.1	4.1	0.9445
315	87.5	88.2	38.4	0.9994	92.7	7.1	0.9232

^a^ Experimental data; ^b^ Calculated value according to Langmuir isotherm model.

**Table 3:** Comparison of the maximum adsorption capacity and selectivity for Pd(II) with other adsorbents at room temperature.

Adsorbent	Capacity (mg·g^−1^)	Selectivity	Refs.
Chitosan	5.88	Not discussed	[16]
MBT-resin	50.0	Not discussed	[29]
Functionalized nanofibers	4.3	Pt(IV) was adsorbed	[30]
Cellulose-MBT	5.00	Zn^2+^, Cd^2+^, Pb^2+^, Hg^2+^ were adsorbed	[31]
carboxymethylchitin hydrogels	2.68	Not discussed	[32]
Chitosan resin	109.5	Pt(IV) and Au(III) were adsorbed	[33]
MFT-resin	15.3	Cu(II) and Zn(II) were adsorbed	[34]
Modified silica gel	77.7	Cu(II), Zn(II), Fe(III) were adsorbed	[35]
α-MnO_2_ nanorods	7.9	Pt(IV), Fe^3+^, Cu^2+^, Ni^2+^, Co^2+^ were adsorbed	Present work
BISIN	81.9	Pt(IV), Fe^3+^, Cu^2+^, Ni^2+^, Co^2+^ were not adsorbed	Present work

**Table 4:** Thermodynamic parameters for the absorption of Pd(II).

Temperature (*K*)	*∆G* (kJ·mol^−1^)	*∆S* (J mol^−1^·K^−1^)	*∆H* (kJ·mol^−1^)
295	−20.1	352.4	83.9
305	−23.6		
315	−27.1		

**Table 5:** Adsorption and separation of palladium from leaching liquor of Cu–Ni sulfide ores.

Metal Ion	Pd(II)	Pt(IV)	Fe^3+^	Cu^2+^	Ni^2+^	Co^2+^
Initial Concentration (mg·L^−1^)	31.5	25.8	108.2	61.7	71.2	47.1
Adsorption rate (%)	99.2	1.99	0.65	0.57	1.26	1.10
Separation factor (β_Pd/M_ × 10^−3^)		6.08	19.2	21.9	9.76	11.0
elution rate (%)	98.5					
Recovery rate	97.7					

**Table 6:** Crystallographic data and refinement parameters for PdCl_2_(S_201_)_2_.

Compound	PdCl_2_(S_201_)_2_
Empirical formula	C10 H22 Cl Pd0.50 S
Formula weight	262.99
Temperature/K	293(2)
Wavelength (Å)	0.71073Å
Crystal system	Monoclinic
Space group	P2(1)/c
Unit cell dimensions (Å, °)	a = 12.0641(13) α = 90 b = 10.9919(12) β = 99.5260(10) c = 10.3523(11) γ = 90
Volume (Å^3^)	1353.9(3)
Z	4
Calculated density (mg/cm^3^)	1.290
Absorption coefficient (mm^−1^)	1.040
F (000)	552
Crystal size (mm^3^)	0.28 × 0.26 × 0.24
θ range for data collection (°)	2.52 to 28.24
Limiting indices	−14 ≤ h ≤ 15, −14 ≤ k ≤ 8, −13 ≤ l ≤ 13
Reflections collected	8889
Independent reflection	3184 [R(int) = 0.0257]
Max. and min. transmission	0.7885 and 0.7595
Refinement method	Full-matrix least-squares on F^2^
Data/restraints/parameters	3184/0/119
Goodness-of-fit on F^2^	1.013
Final R indices [I > 2σ(I)]	R1 = 0.0298, wR2 = 0.0710
R indices (all data)	R1 = 0.0490, wR2 = 0.0797
Largest diff. peak and hole (Å^−3^)	0.339 and −0.307

**Table 7:** Crystallographic data and refinement parameters for thiourea-Pd(II) complex.

Compound	Thiourea-Pd(II)
Empirical formula	C_4_H_16_Cl_2_N_8_PdS_4_
Formula weight	481.79
Temperature/K	298(2)
Wavelength (Å)	0.71073
Crystal system	Orthorhombic
Space group	Pna2(1)
Unit cell dimensions (Å, °)	a = 12.9203(10) α = 90 b = 8.2613(7) β = 90 c = 15.1500(12) γ = 90
Volume (Å^3^)	1617.1(2)
Z	4
Calculated density (mg/cm^3^)	1.979
Absorption coefficient (mm^−1^)	1.993
F (000)	960
Crystal size (mm^3^)	0.20 × 0.18 × 0.16
θ range for data collection (°)	2.69 to 28.31
Limiting indices	−16 ≤ h ≤ 17, −10 ≤ k ≤ 9, −20 ≤ l ≤ 19
Reflections collected	10235
Independent reflection	3750 [R(int) = 0.0253]
Max. and min. transmission	0.7410 and 0.6913
Refinement method	Full-matrix least-squares on F^2^
Data/restraints/parameters	3750/1/172
Goodness-of-fit on F^2^	1.116
Final R indices [I > 2σ(I)]	R1 = 0.0250, wR2 = 0.0541
R indices (all data)	R1 = 0.0296, wR2 = 0.0652
Largest diff. peak and hole (Å^−3^)	0.349 and −0.711
